# Being pragmatic about healthcare complexity: our experiences applying complexity theory and pragmatism to health services research

**DOI:** 10.1186/s12916-018-1087-6

**Published:** 2018-06-20

**Authors:** Katrina M. Long, Fiona McDermott, Graham N. Meadows

**Affiliations:** 1 Southern Synergy, Department of Psychiatry, School of Clinical Sciences at Monash Health, Faculty of Medicine, Nursing and Health Sciences, Monash University, Melbourne, VIC Australia; 2 Department of Social Work, School of Primary and Allied Health Care, Faculty of Medicine, Nursing and Health Sciences, Monash University, Melbourne, VIC Australia; 3 Melbourne School of Population and Global Health, University of Melbourne, Melbourne, VIC Australia; 4 Monash Health, Melbourne, VIC Australia

**Keywords:** Complexity theory, Pragmatism, Health services research, Epistemology, Methodology, Implementation science

## Abstract

**Background:**

The healthcare system has proved a challenging environment for innovation, especially in the area of health services management and research. This is often attributed to the complexity of the healthcare sector, characterized by intersecting biological, social and political systems spread across geographically disparate areas. To help make sense of this complexity, researchers are turning towards new methods and frameworks, including simulation modeling and complexity theory.

**Discussion:**

Herein, we describe our experiences implementing and evaluating a health services innovation in the form of simulation modeling. We explore the strengths and limitations of complexity theory in evaluating health service interventions, using our experiences as examples. We then argue for the potential of pragmatism as an epistemic foundation for the methodological pluralism currently found in complexity research. We discuss the similarities between complexity theory and pragmatism, and close by revisiting our experiences putting pragmatic complexity theory into practice.

**Conclusion:**

We found the commonalities between pragmatism and complexity theory to be striking. These included a sensitivity to research context, a focus on applied research, and the valuing of different forms of knowledge. We found that, in practice, a pragmatic complexity theory approach provided more flexibility to respond to the rapidly changing context of health services implementation and evaluation. However, this approach requires a redefinition of implementation success, away from pre-determined outcomes and process fidelity, to one that embraces the continual learning, evolution, and emergence that characterized our project.

## Background

Complexity theory has become increasingly popular in healthcare research over the last two decades. Its emergence provides credence to the growing arguments from health services researchers, namely that the healthcare system contains a level of complexity qualitatively different to other systems due to the social nature of health, and therefore requires a different set of research methods [1–4]. Indeed, the mismatch between this hypercomplexity [1] and the dominant mechanistic conception of healthcare [5, 6] has been used as a key explanation for the lack of implementation of evidence-based medicine [4, 7] and healthcare innovation [6, 8–10].

Herein, we discuss our recent experiences implementing and evaluating healthcare simulation modeling in a large Australian health service. We first explore some of the contributions of complexity theory to our understanding of the healthcare context, reviewing some of the key debates in this emerging field. We then explore the possibilities of using pragmatism to provide the missing epistemological foundation required to structure the study of social complexity theory in healthcare. Finally, we revisit our case study to discuss how we put pragmatic complexity research into action as the evaluation framework for a simulation modeling project.

## The real world of healthcare implementation and evaluation

We begin by describing the initial aims and scope of the healthcare simulation modeling project, which provides the practical case study for this article. We will return to the case study throughout the article to demonstrate some of our theoretical arguments. The case study discussed here presents the researcher experience of the implementation process, which ultimately shaped and motivated the epistemological quest that is the subject of this paper. For readers interested in the outcomes of this project, we refer them to our upcoming evaluation paper, which provides a more detailed account of the application of the epistemology, theory, and methods discussed in this article to implementation evaluation.

The 3-year research project aimed to develop simulation models to provide strategic decision support for a senior leadership group (SLG) in a large public mental health service (MHS) in Australia. The MHS was responsible for government-funded inpatient and community mental health services across the age spectrum, with different but overlapping catchment areas for early in life (under 25 years), adult, and aged (over 65 years) services.

The project was designed to consist of four major phases, namely (1) the development of a conceptual framework for the simulation model, (2) integration with simulation software, (3) validation of the model, and (4) implementation of the model within the MHS as a decision support tool. The initial scope included a model of the whole MHS, with the option of additional scenarios of specific interest to managers. Three general types of scenarios were identified at the outset, namely (1) policy change affecting the structure of services, (2) population distribution changes, and (3) organizational innovation in delivery of care models. The planned modeling approach was discrete-event simulation using the ARENA® software package [11].

The original project research team included nine investigators with experience in psychiatric epidemiology, health economics, simulation modeling, health services planning, organizational change management, action research, and qualitative methodologies. Two of these researchers were existing members of the SLG, and brokered research access with the MHS.

A key feature of the project, as planned, was the involvement of the SLG throughout the project via regular presentations and workshops at the existing monthly SLG meetings. Involvement of the SLG was intended as essential in generating scenarios for simulation, developing clinically accurate conceptual models of patient transitions (within the MHS), and validating the model for use by decision-makers. However, about a year into the project, the MHS underwent a major restructuring after a significant number of senior staff left the service. The decision-making processes in the organization changed substantially so that simulation-related interactions between researchers and stakeholders became more reliant on one-on-one and small group discussions. At the same time, changes in policy, such as the introduction of the Victorian Mental Health Act [12] and National Disability Insurance Scheme Act [13], and a freeze or contraction in state and federal mental health funding [14, 15], changed the strategic priorities and decision-making scope of the MHS. Consequently, some simulation models in development were no longer of immediate relevance to the participants, while other issues that came to the fore, such as the redrawing of clinical catchment areas, did so with time-decision horizons not compatible with the development time costs of discrete event simulation. The researchers adapted, changing both their methods and focus to align with the new strategic directions and concerns of the service.

This experience may be familiar to many implementation scientists and healthcare managers; however, it does pose significant challenges for evaluators. We subsequently outline how, by applying the twin lenses of complexity theory and pragmatism, we developed a deeper understanding of the processes of implementation.

## A view from complexity theory

### A health services research project as a complex adaptive system (CAS)

There is no doubt that the context described above is complex, or in the language of complexity theory, a CAS [16]. A CAS is formally defined as “*a collection of individual agents with freedom to act in ways that are not always totally predictable, and whose actions are interconnected such that one agent’s actions change the context for other agents*” ([5], p. 625). While there is still some disagreement over the terminology, the key features of a CAS generally include embeddedness, nested systems, fuzzy boundaries, distributed control, self-organization, emergence, unpredictability, non-linearity, phase changes, historicism, sensitivity to initial conditions, non-equilibrium, adaptation, and co-evolution (Box 1) [4, 8, 9, 17–19].

Many of these features of a CAS were found in our experiences. The project involved multiple nested systems, namely the researcher group, SLG, MHS, and the state and federal governments. Boundaries between systems were fuzzy, with participants often exerting influence in multiple systems. For example, in addition to their employment with the MHS, a significant number of SLG participants held roles within university departments, government advisory boards, discipline-specific associations (e.g., Royal Australian and New Zealand College of Psychiatrists, Australian Medical Association, Australian Psychological Society), or private consulting clinics. Control was distributed, with members of the SLG holding responsibility and autonomy over the running of different programs within the MHS, researchers exerting control over the implementation activities, and politicians, bureaucrats, and senior healthcare managers making policy and funding decisions that affected the operating context of the SLG and researchers.

Changes in the SLG were unpredictable and non-linear, instead emerging from what may be considered phase changes in the system. For example, the first restructure of the SLG did not occur until a key influential member had been convinced of its merit. It was only with the support of this individual that the change occurred, representing a phase change in the organizational context. This started a period of 2 years of continual staff and role changes within the SLG, which could not have been foreseen by the individual whose support initiated the process. Both the SLG and the researchers exhibited adaptation and co-evolution, changing strategic priorities and approaches based on the changes in context. For example, as the state government signaled an increased interest in infrastructure planning for population growth through a series of discussion papers, the researchers refocused their modeling efforts on the area. When new mental health funding was released by the state government in 2017, members of the SLG, aware of the researcher activity in this area, successfully lobbied for funding based on this modeling output.

### Applying complexity theory to healthcare research

Rather than attempting to control the research context, complexity theory directs researchers to make it the focus of their study, looking for patterns of interactions within agents, and between agents and the environment to explain system-level outcomes [17]. In healthcare, these systems level outcomes represent public health interests such as the efficiency and effectiveness of healthcare delivery, the population’s quality of life, and rates of disease morbidity and mortality. Embracing this approach removes the focus from the short-term outcomes of individual interventions (often randomized control trials), which are isolated from the rest of the healthcare system, and places it on understanding the complex contextual factors that determine the long-term survival of a new healthcare intervention.

A classical approach to complexity theory directs researchers to identify rules that govern these behaviors, attributing them to the agent (local rules) or an environmental pattern (attractors). In this classical interpretation of complexity theory, established research methods include agent-based modeling, simulation, and network analysis, where a theory of local rules is built into a mathematical model, which is tested against reality [17, 19–21]. However, these approaches have had limited success in healthcare, with low rates of modeling implementation [22–24] often being attributed to the lack of good data from which to build models [25–28]; the complex social and organizational context of healthcare, with multiple intersecting and nested stakeholder groups [1, 2, 25, 27, 29, 30]; and the high expertise and time costs of creating sufficiently complex, ecologically valid models [25–29, 31–33].

Recent applications of complexity theory to healthcare have branched out into more qualitative methods, including ethnography, case studies, case–comparison or time-series analyses, and social surveys [10, 18, 20, 34]. These approaches emerged from the seminal work of Byrne [18], who translated many of the concepts of complexity theory into the social realm.

## Debates in social complexity theory

Complexity theory has alternatively been defined as a methodology [35], conceptual framework [4, 20, 36], metaphor [4, 34], world view [37], frame of reference [34], ontology [34], or as a “*loose set of concepts, heuristics, and analytic tools*” ([8], p. S31). Different authors have posited different typologies of complexity science to address this lack of coherence (e.g., [1, 8, 18, 38]), with a clear delineation emerging between the complexity theory of things (classic complexity theory, e.g., cells, animals, atomic particles) and that of humans (social complexity theory). The multitude of actors, motives, and behaviors animating social complexity theory poses significant challenges to both theorizing and researching. Below, we outline the key tensions in this emerging field.

### Description or explanation?

In his critique of social complexity theory, Paley states that “*complexity is an explanatory concept*” ([39], p. 59). Social complexity theorists seem to disagree, describing emergence as descriptive, not explanatory [40], and arguing that the only way to see the outcome of a CAS is to observe the system as a whole, rather than its component individual agents or models [5]. This raises the fundamental issue of epistemology. The classical complexity theory focus on explanation aligns with a positivist epistemology, where knowledge is valued if it is generalizable and allows us to predict, and manipulate, future behavior [25]. This clearly aligns with the aim of implementation and most public health research, which is namely to affect meaningful change. The epistemology of social complexity theory, on the other hand, is unclear. If social complexity theory does represent a purely descriptive epistemology, which makes no claims to the translation of findings across contexts, then its ability to contribute to implementation science may be minimal.

### The role of agency in self-organization

The redefinition of local rules as human instincts, constructs, and mental models has also been subject to debate [5, 39]. This is in part due to the inherent problems with trying to measure internal states, with even qualitative methods heavily reliant on individual insight and candor [21]. This is also due to the lack of fit between the focus of classic complexity theory, individual agent survival, and the postmodern ideas of democracy and collectivism which shape the social world. While survival in biological systems can be treated as a key driver and outcome measure, the survival of organizations, systems of operations, and even ideas are less necessary, or observable, in social systems [19]. This creates a rift in complexity theory’s understanding of agency. Classic complexity theory defines agency as an agent’s enactment of their local rules, which ensures their survival, while social complexity theory defines agency as the cognitive, motivational, and emotionally driven intentional behaviors that agents employ to achieve their end goal. This end goal is not always individual survival. Given that Byrne et al. [20] identified agency as a key defining feature of social complexity theory, and a key target for research, how are we to study it, considering these definitional issues?

We found this to be a key challenge in our project. In order to evaluate the effect of the simulation modeling on the decision-making processes of the SLG, we attempted to use interviews to establish a baseline picture of the relationships, mental models, and expectations of the individual participants. However, the experience of interviewing suggested that disclosure levels varied significantly between participants, influenced by their perceptions of the vested interests of the research group, and the existing personal relationships between researchers and SLG members. We also faced difficulties in that time and access limitations of working with senior managers often meant that data were not collected when significant decisions were made or events occurred. We therefore had to rely on the retrospective recall of participants to piece together a picture of the events, and their roles in them. This approach meant that our image of individual events was often incomplete, preventing us from accurately identifying the role of individual agency in the observed interactions and system-level changes.

### Defining social CASs

There are two pervasive issues with defining a social system, nesting and fuzzy boundaries, both of which are implicated in, and complicate, complexity research [19]. In the health system, Byrne et al. [20] identified four levels of nested systems, namely the individual, population health, the health service system, and the planetary ecosystem. However, several more exist within the health service system, including general practices, practice networks, hospitals, hospital networks, and national programs [5]. Thus, a key question facing complexity researchers is which systems should form the core of the analyses, and how many levels of analysis are sufficient to provide a complete understanding of the system.

The boundaries of social systems are also harder to define and control than in a classic CAS [21, 34]. As we discovered in our efforts to develop simulation models of mental health patients, a patient may pass through multiple different practices, hospitals, and even districts over an episode of care, interacting with scores of individual agents, each operating in a different context. Likewise, the boundaries of the implementation context proved hard to define. Despite beginning with a focus on the MHS as the key implementation context and the SLG as the key agents, it emerged through the course of the evaluation that the context of individual researchers (e.g., contract changes, relocations, life events), researcher team dynamics, and the wider government contexts exerted very significant influences on the trajectory of the project. Thus, system boundaries are often arbitrary, with implementation and evaluation researchers required to balance descriptive sufficiency with practicality.

These issues lead us to a key consideration – in light of these debates in social complexity theory, how can complexity researchers make transparent and consistent decisions regarding research methodology. While social complexity theory offers a clear ontology, focusing on agent interactions and emergent system outcomes [34], it lacks a clear position on the epistemic contribution of studying CASs. We suggest that what is needed is a clear epistemology [4], and we suggest that pragmatism may provide the epistemological foundations required to structure the study of social complexity theory in healthcare.

## A contribution from pragmatism

### What is pragmatism?

We suggest that many healthcare workers would identify as pragmatists. The everyday use of the term pragmatism implies a focus on the practical and achievable, rather than the theoretical or ideal [41]. This idea of valuing the applied over the theoretical is mirrored in the philosophy of Pragmatism.

Pragmatism emerged in the late 1800s in the work of Charles Pierce, William James, and John Dewey. At the center of pragmatism is a rejection of the ‘impossible question’ of philosophy, that of the nature of the mind’s relationship to reality [42]. Instead, pragmatists judge the value of knowledge (and our ways of knowing) by its context-dependent, extrinsic usefulness for addressing practical questions of daily life [43]. Perfect knowledge is not possible, nor required. For pragmatism, knowledge is only meaningful when coupled with action [38].

There are many similarities between the arguments of social complexity researchers and pragmatists. Below we explore key synergies (Box 2).

### Contextualized research

A key feature of pragmatism is the contextualization of knowledge [44, 45]. As contexts change, so too do the criteria of usefulness for knowledge. Similarly, social complexity theory calls for the matching of research approach to context and level of environmental complexity [4, 9]. In complexity theory, these contexts could include different nested systems, and different time points [44]. Therefore, in order to maintain a coherent research agenda in a CAS, a unifying research question is required.

In our project, the response to the challenge of working within this particular CAS manifested through the emergent formulation of two deeply pragmatic research questions: How can we (the researchers) help to improve strategic decision-making for mental health services? What can we learn of value through this process? This allowed us, as the context changed, to maintain the same focus for the project, but change and expand the evaluation focus from the experiences of the SLG to include, for instance, adaptations of the researchers to the changing stakeholder needs. The same aims were addressed, but using different methods.

### Continual learning

The contextualization of knowledge does not reject the translation of knowledge between contexts. While pragmatism does hold that knowledge is not completely generalizable, it also argues that imported knowledge can play a role in shaping observation and perception and in suggesting possible solutions to the current problem [42]. For implementation science, the merging of complexity theory’s deep focus on contextual interactions and emergent outcomes, coupled with pragmatism’s perspective on knowledge translation, provides a way of fostering collective implementation learning [16, 46], without bowing to the need for research generalizability.

For our project, this led us to re-define implementation success, not as a strict adherence to the project plan or the achievement of pre-determined outcomes (i.e., the publication of four simulation models and the use of these models to inform decisions), but by the perceived usefulness of the project to the stakeholders and the lessons learned. As Byrne commented: “*The point about complexity is that it is useful – it helps us to understand the things we are trying to understand*” ([18], p. 7). Indeed, what we learnt was that the simulation models themselves seemed not to be the main outcome of interest to the SLG; instead, it was the personal insights that members gained from the conceptual development discussions and our presentations of amalgamated patient data.

### Research as social action

Another key pillar of pragmatism is the active and social nature of inquiry. Dewey argued that the primary function of research is to solve societal problems [38]. However, he also argues for flexibility in application, proposing “*that policies and proposals for social action be treated as working hypotheses, not as programs to be rigidly adhered to and executed*” ([47], pp. 151–2).

These sentiments are echoed in social complexity theory:

“*Complexity/chaos offers the possibility of an engaged science not founded in pride, in the assertion of an absolute knowledge as the basis for social programs, but rather in a humility about the complexity of the world coupled with a hopeful belief in the potential of human beings for doing something about it*.” ([18], p. 45).

Not only does pragmatism argue for a problem-solving approach to inquiry, but also to an action-based one. All modes of experience, including research, are treated as interventions [42]. Research success within a pragmatic epistemology is measured by consequences, whether they be predicted or emergent. This aligns with the holistic system view of complexity theory, where outcomes are not pre-determined, but emergent [36]. Thus, complexity theory provides a way of operationalizing the study of emergent consequences, while pragmatism provides the impetus for change by measuring research quality with respect to its impact on social change.

### Valuing of different knowledge

The usefulness of knowledge metric also creates a democratization of scientific endeavor. Scientific knowledge is treated not as a qualitatively different form of knowledge, but simply as a more formalized version of everyday human inquiry [48]. Science therefore becomes a social pursuit, within anyone’s reach. This idea of intuitive inquiry aligns with a theme, advanced by many scholars advocating for complexity theory in healthcare, that social actors already have an intuitive sense of complexity, which can be refined by the framework of complexity theory [4, 9]. Social complexity theorists also argue for a natural fit between complexity approaches and participatory research, where participant and researcher frames of reference are treated as equally important to inquiry [20], failure is tolerated and expected [49], and innovation is allowed to emerge from any part of the system [9].

In our project, this led to a fundamental shift in the implementation evaluation from a focus purely on the participant experience, to one that included the experiences of the researchers. In the initial design of the evaluation, the CAS of interest was that of the SLG. Our evaluation was focused on understanding the decision-making mental models of these individuals, and how they negotiated shared group processes and behaviors based on these individual models. However, the organizational restructure of the SLG affected not only access to participants for evaluation data collection, but also affected the researchers’ approach to the simulation modeling development and implementation. As mentioned above, one way this manifested was as a change in engagement with members of the SLG. Researchers began using one-on-one interactions with engaged SLG members to develop new scenarios directly related to the SLG members’ portfolio. Therefore, the experiences and reflections of the researchers became pivotal in understanding the project’s implementation after the organizational restructure.

Both pragmatism and complexity theory also encourage a focus on the interactions of knowledge systems, and the study of how these intersections are negotiated [4, 44, 48]. For us, this manifested as multiple themes emerging from a grounded theory approach to the implementation evaluation, including participant-researcher communication (frequency, modality, content), understanding and expectations of the modeling methodology, and different outcome priorities between the researchers and participants. The case study approach of the evaluation, supported by interviews and unstructured observation, allowed these themes to emerge, but there remains a challenge for creating more targeted research designs and methods capable of capturing, measuring, and interpreting these interactive and emergent processes.

### Support for mixed methods research

A key theme in the development of social complexity research is the call for mixed methods research [8, 34]. However, there is a risk of method choice being driven by the ‘what works’ maxim [50]. As one of the key epistemologies for mixed method research, pragmatism offers a more structured approach to mixed methods research [42]. Pragmatism calls for choices of research questions and methods to be driven by the social purpose of the research, not the other way around [42, 45, 51].

Another of the risks identified by complexity theorists is the pre-emptive labelling of a system as complex [40]; a pragmatic approach does not require such a priori assumptions. Rather, it allows for the flexible use of multiple methods to capture insights in a complex environment, which may later be interpreted using a range of frameworks. Therefore, our pluralism of evaluation methods (i.e., interviews, questionnaires, document analysis, observations) provides us with multiple perspectives to be explored and structured in different ways in order to ultimately build an understanding of the process of implementation.

Pragmatism also encourages reflection and experimentation, allowing for the evolution of interventions and evaluation in a similar fashion to a CAS [7, 42, 45]. Therefore, our shift in evaluation from the quantitative analysis of participant questionnaire responses to a grounded theory case study of research adaptation is not only consistent with complexity theory, but predicted by it, as a co-evolution of the researchers in context. Thus, rather than rejecting the reductionist approach of classic complexity theory [20], pragmatism allows for the contribution of both quantitative and qualitative methods in addressing the research question. It also allows for different definitions of complexity theory. Complexity theory can be both an ontology for quantitative approaches and a metaphor for qualitative approaches.

## The case study revisited

Our case study illustrates how a pragmatic epistemology can support, and broaden, the application of complexity theory to healthcare implementation and evaluation.

By starting from a pragmatic epistemology, we allowed our focus to be drawn to the most relevant ontology and methodologies for the study of this implementation. Complexity theory emerged as a relevant theory and ontology for the analysis; however, we do not hold that it is the only possible lens through which to evaluate the implementation. A pragmatic frame encouraged us to embrace different types of inquiry and data collection methods, using questionnaire, interview, observation, and document analysis approaches. As the implementation progressed, we included new participants (i.e., researchers), and expanded our frame of data collection to include government policy and funding changes. By doing so, we overcame one of the key challenges in social complexity theory – defining the CAS of interest.

In our evaluation, we pragmatically allowed implementation success to be defined by the collection of stakeholders, honoring the multitude of different expectations held by the research funding body, the academic community, and individual members of the SLG and research team. We then began the data analysis with a critical incident approach to identify turning points in the system, which were investigated further with thematic analysis. It was only when the emerging themes resonated with a complexity theory interpretation of the project that we labeled our case study as a healthcare implementation CAS.

## Conclusions

Herein, we described a too-familiar experience in health services implementation – a constantly changing implementation context– followed by a discussion of how complexity theory and pragmatism provide complementary approaches to the difficulties in evaluating such implementations. The commonalities between pragmatism and complexity theory are striking, and include a sensitivity to research context, a focus on applied research, and the valuing of different forms of knowledge. For implementation and evaluation, this fusion of approaches has significant implications:

- A focus on researcher and stakeholder agency, in shaping the direction and outcomes of interventions.
- A re-definition of implementation success, not as a strict adherence to the project plan, or the achievement of pre-determined outcomes, but as the emergent outcomes of the project and lessons learned, as identified by all stakeholders.
- A flexibility in implementation and evaluation methods, encouraging the reflexive use of mixed methods to capture and adapt to the changing research context.
- A rejection of the description-explanation divide, focusing instead on continual, collective learning, where case studies provide starting points, not theories, for future research.

However, our recommendations are not without limitations. There are other epistemic options for complexity theory, including nested theories [34], an eclectic use of middle-range theories [37], or a pluralistic ontology of levels supported by emergence [26]. One of the more promising alternatives comes from Byrne et al.’s [20] application of complex realism to complexity theory. At face value, the arguments of complex realism seem not incommensurate with pragmatism [42]; however, we will leave a detailed comparison of these two approaches to future scholars. Alternatively, complexity theorists may entirely reject our suggestion of the need for an epistemology. Another limitation is posed by the theoretically agnostic position of pragmatism, as outlined above. It is highly likely that a pragmatic approach will not always support the application of complexity theory in healthcare implementation research. While we believe this is a strength in the use of pragmatism in healthcare implementation, it may limit the uptake of pragmatism by researchers who specialize in complexity theory.

The application of complexity theory to social science, including healthcare, is still in its infancy. So too is the formalization of pragmatism as a school of philosophy [43]. However, we agree with Talisse and Aikin, in that discussions such as those presented in this article are a positive sign, “*a mark of …vitality, an indication that it is a living philosophy rather than a historical relic.*” ([43], p. 3). We present this article in that spirit and hope that our contribution sparks further discussion about the potential collaboration of pragmatism and complexity theory in informing implementation science and health services research.

## Box 1. Key features of complex adaptive systems (CASs)

**Embeddedness/nested systems:** CASs are embedded within a wider context and other CASs.

**Fuzzy boundaries:** System boundaries are permeable and hard to define.

**Distributed control and self-organization:** System patterns are not created by top-down control; instead, autonomous agents interact to create outcomes. Thus, organization in a CAS emerges naturally from local rules held by agents.

**Emergence:** Interactions between agents create system outcomes that are not directly intended and are greater than the sum of the individual agent behaviors.

**Unpredictability:** The behavior of a CAS cannot be predicted due to its non-linearity, sensitivity to initial conditions, and historicism.

**Non-linearity:** The magnitude of system input and agent interactions is not linearly related to the magnitude of changes in the system. A CAS can react suddenly to minor inputs or fail to change despite overwhelming external pressure.

**Phase changes:** Where a small change in the system inputs results in a qualitative change in the system’s state.

**Sensitivity to initial conditions and historicism:** Future agent actions are affected by past changes in the system, leading initial conditions to exert a strong influence on system behaviors.

**Non-equilibrium:** CASs are characterized by continual change and do not reach equilibrium.

**Adaptation and co-evolution:** Agents and systems evolve together, reacting to changes in the context to ensure optimal functioning and survival.

## Box 2: Similarities between social complexity theory and pragmatism

Both:

- Aim to create ‘useful’ knowledge
- Reject reductionist science in favor of the study of whole systems, in context
- Understand research as a continual learning process
- Focus on the social consequences of research and intervention
- Value the democratization of knowledge and research, valuing all stakeholders’ input
- Prioritize understanding over theoretical or methodological purity, encouraging the use of multiple methods

## Abbreviations

CAS: complex adaptive system
MHS: mental health service
SLG: senior leadership group
